# MiR-3622a-3p acts as a tumor suppressor in colorectal cancer by reducing stemness features and EMT through targeting spalt-like transcription factor 4

**DOI:** 10.1038/s41419-020-02789-z

**Published:** 2020-07-27

**Authors:** Shuchen Chang, Guangli Sun, Dan Zhang, Qing Li, Haihua Qian

**Affiliations:** 1 Department of Anorectal Surgery, The Affiliated Hospital of Nanjing University of Chinese Medicine, Nanjing, 210029 Jiangsu province China; 2 Department of General Surgery, The First Affiliated Hospital of Nanjing Medical University, Nanjing, 210029 Jiangsu province China; 3 Medical College of Southeast University, Nanjing, 210009 Jiangsu province China

**Keywords:** Colorectal cancer, miRNAs

## Abstract

MicroRNAs are a class of small non-coding RNAs which act as oncogenes or tumor suppressors through targeting specific mRNAs. Colorectal cancer (CRC) is one of the most common malignancies worldwide. MiR-3622a-3p is found to be decreased in colorectal cancer (CRC) by analyzing data from TCGA database and there are few reports about the role of miR-3622a-3p in cancers. Our research aimed to explore the effects of miR-3622a-3p on CRC. MiR-3622a-3p was found to be down-regulated in CRC tissues and cells by qRT-PCR. The effect of miR-3622a-3p on proliferation, apoptosis, cell cycle, migration and invasion of CRC cells were investigated by a serious of biological function assays and the results revealed that miR-3622a-3p could inhibit the malignant biological properties of CRC. We performed dual luciferase assay, RNA immunoprecipitation (RIP) assay and pull-down assay to confirm the interaction between miR-3622a-3p and spalt-like transcription factor 4 (SALL4). Western blot was carried out to determine the effects of miR-3622a-3p and SALL4 on stemness features and EMT. We found that miR-3622a-3p suppressed stemness features and EMT of CRC cells by SALL4 mRNA degradation. MiR-3622a-3p could inhibit CRC cell proliferation and metastasis in vivo with tumor xenograft model and in vivo metastasis model. The CRC organoid model was constructed with fresh CRC tissues and the growth of organoids was suppressed by miR-3622a-3p. Taken together, the results of our study indicate miR-3622a-3p exerts antioncogenic role in CRC by downregulation of SALL4. The research on miR-3622a-3p might provide a new insight into treatment of CRC.

## Introduction

Colorectal cancer (CRC) remains to be one of the most common malignancies all over the world^1^. It was estimated that more than 1.8 million new CRC cases occurred and 881000 CRC patients died in the year of 2018^2^. The incidence and mortality of CRC ranked the fourth and the fifth, respectively, in China^3^. Treatment methods for CRC include surgery, chemotherapy and radiotherapy. Despite great improvement has been made in diagnosis and treatment for CRC, the 5-year survival rate is still low and varies a lot based on the specific clinical stages^4,5^. One of the reasons for CRC patients’ poor prognosis is lack of early diagnosis biomarkers and effective treatment targets. It is urgently required to uncover the molecular mechanisms underlying CRC.

MicroRNAs (miRNAs) are a class of small non-coding RNAs which consist of 20–24 nucleotides and can regulate expression of targeted genes by binding to their 3′-untranslational regions (3′-UTR)^6,7^. MiRNAs have been demonstrated to play a promotive or inhibitory role in many types of tumors^8^. MiR-3622a-3p has been reported to promote development of bladder cancer by targeting LASS2^9^. It has also been reported that miR-3622a-3p could suppress prostate cancer progression by inhibiting epithelial–mesenchymal transition (EMT)^10^. However, the effect of miR-3622a-3p on CRC has not been elucidated so far.

Spalt-like transcription factor 4 (SALL4) is one of the members of SALL gene family. It functions as a zinc finger transcription factor and maintains pluripotency of embryonic stem cells (ESCs) by regulating Nanog, Sox2 and Oct4^11–13^. SALL4 has been first reported to be abnormally expressed in human acute myeloid leukemia (AML) and regulate survival and apoptosis of leukemic cells^14,15^. In addition to AML, SALL4 is also found to function in solid tumors. SALL4 promotes invasion capacity of EpCAM-positive hepatocellular carcinoma by regulating stemness^16^. SALL4 could induce EMT and chemoresistance in endometrial cancer^17^. Overexpression of SALL4 contributes to tumor growth in breast cancer^18^. In gastric cancer, SALL4 is a biomarker for tumorigenesis and metastasis^19^. Knockdown of SALL4 inhibits CRC carcinogenesis and SALL4 could be a critical biomarker for screening of early CRC patients^20,21^.

The existence of cancer stem-like cells (CSCs), which contribute to tumor initiation, proliferation and migration is considered to be one of the barriers for treatment of cancers, including CRC^22,23^. The presence of CSCs also accounts for tumor drug resistance and reoccurrence^24^. EMT means a process by which epithelial cells are changed into the cells with stromal properties^25^. Tumor cells which have undergone EMT have increased migratory and invasive properties and become more resistant to apoptosis^26^. The Wnt/beta-catenin signaling pathway has been considered to be conserved during evolution and associated with various processes, including initiation, proliferation, apoptosis, senescence, differentiation and metastasis of tumor cells^27,28^. Activation of Wnt/beta-catenin signaling pathway has been reported to induce EMT, resulting in loss of cell-cell adhesion^29^. In addition to EMT, Wnt/beta-catenin signaling has also been verified to promote cancer stemness features of CRC^30^.

In our research, we studied the biological functions of miR-3622a-3p and the underlying molecular mechanism. The results obtained from our study support the hypothesis that miR-3622a-3p suppresses progression and metastasis of CRC by SALL4 mRNA degradation and inactivation of Wnt/beta-catenin signaling pathway.

## Results

### MiR-3622a-3p was down-regulated in CRC

**Fig. 1:**
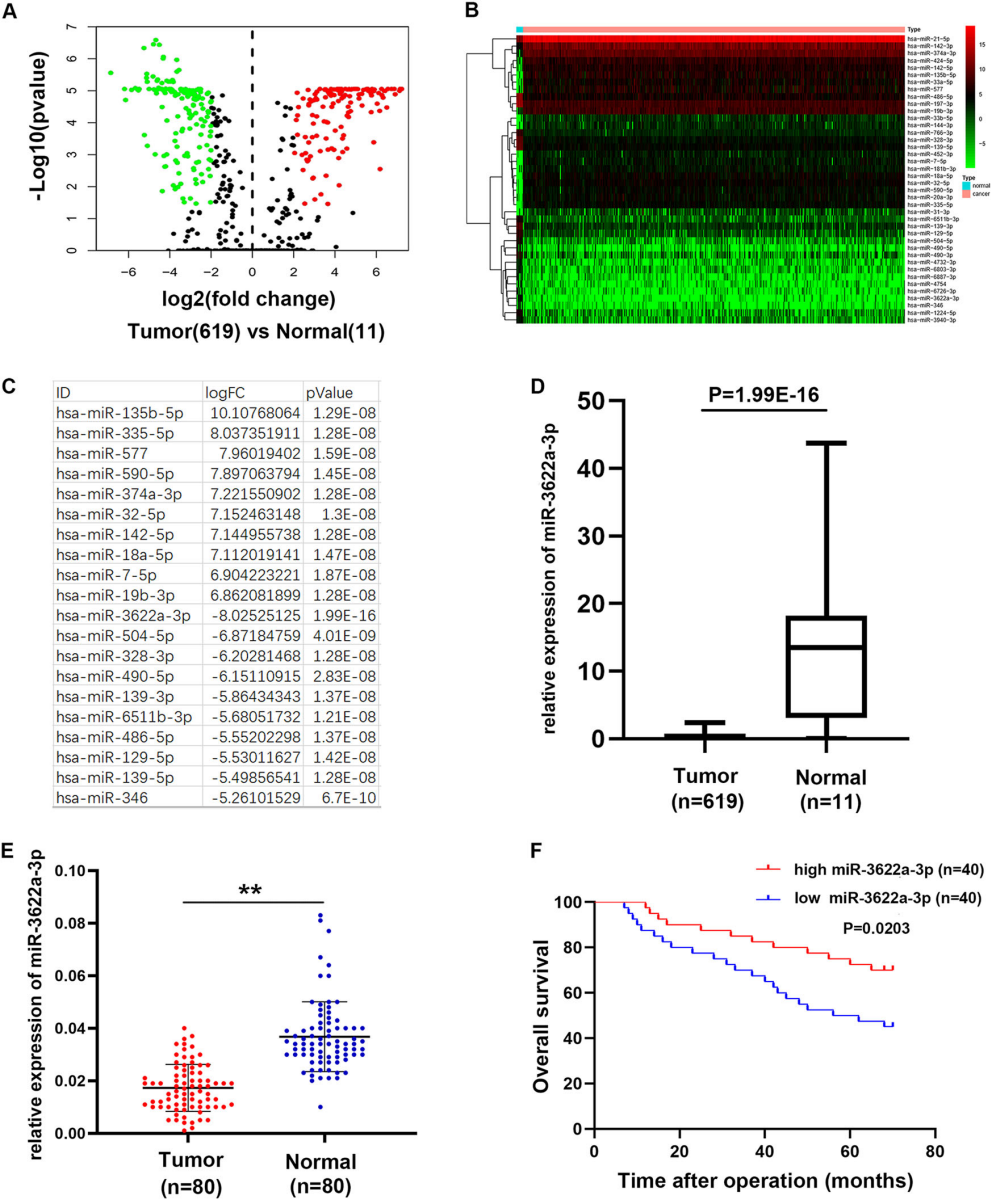
MiR-3622a-3p was identified to be down-regulated in CRC. **a** The volcano plot of the differentially expressed miRNAs in CRC from TCGA database. **b** The cluster heat map of the top 20 up-regulated and down-regulated miRNAs in CRC from TCGA database. **c** The top 10 up-regulated and down-regulated miRNAs in CRC were listed. **d** MiR-3622a-3p was the most down-regulated miRNA in CRC based on the analysis of TCGA miRNAs-seq data. **e** MiR-3622a-3p was confirmed to be down-regulated in CRC by qRT-PCR performed on 80 pairs of CRC tissues and adjacent normal tissues. **f** Overall survival (OS) analysis revealed that high miR-3622a-3p expression was an advantage for CRC patients’ prognosis. All data are from three independent experiments and are presented as the means ± SD (*p < 0.05, **p < 0.01).

To investigate the miRNA expression pattern in CRC, the miRNAs-seq data which consist of 619 CRC specimens and 11 normal specimens was downloaded from The Cancer Genome Atlas (TCGA) database. The differentially expressed miRNAs were shown in the volcano plot (Fig. 1a). 141 miRNAs were up-regulated in CRC (Log2 FC > 2, *p* < 0.05) while 171miRNAs were down-regulated in CRC (Log2 FC < −2, *p* < 0.05). The top 20 up-regulated and down-regulated miRNAs were presented in the cluster heat map (Fig. 1b). As shown in Fig. 1c, the top 10 up-regulated and down-regulated miRNAs were listed. Among all the differentially expressed miRNAs, we found miR-3622a-3p was the most down-regulated miRNA (Fig. 1d). Then we detected miR-3622a-3p expression in 80 pairs of CRC tissues and adjacent normal tissues. As shown in Fig. 1e, miR-3622a-3p was obviously down-regulated in CRC tissues. The 80 CRC patients were divided into high group and low group based on miR-3622a-3p expression. We analyzed the relationship between clinicopathological features of the CRC patients and miR-3622a-3p expression level. As shown in Table 1, miR-3622a-3p expression was negatively corelated with tumor size and lymph node metastasis. The CRC patients with high level of miR-3622a-3p expression had better prognosis compared with the low miR-3622a-3p expressed CRC patients (Fig. 1f). DNA methylation has been validated to regulate expression of miRNA genes in different kinds of tumors^31–33^. Pri-miR-3622a-3p, pre-miR-3622a-3p and mature miR-3622a-3p were examined by qRT-PCR in CRC cells after treatment of 5-aza-2′-deoxycytidine (5-Aza), which is a DNA methyltransferase inhibitor. As shown in Supplementary Fig. 1A, pri-/pre- and mature miR-3622a-3p was significantly up-regulated by 5-Aza treatment. It was also observed in Supplementary Fig. 1B that pri-/pre- and mature miR-3622a-3p increased obviously after treatment with trichostatin A (TSA), an inhibitor of the class I and II HDAC families. We used an online software (www.urogene.org) to analyze the genomic locus of miR-3622a-3p and found that there was a region with high frequency of CpG sites (Supplementary Fig. 1C). Then bisulfate genomic sequencing (BSP) assay was performed and the results indicated that methylation level of this region in miR-3622a-3p promoter was elevated in CRC cells compared with human normal colon epithelial cell line NCM460. Taken together, down-regulation of miR-3622a-3p in CRC was controlled by methylation in the promoter of miR-3622a-3p.

**Table 1:** Expression of miR-3622a-3p and SALL4 in human colorectal cancer and the patients’ clinicopathological characteristics.

Characteristics	Number	miR-3622a-3p expression		*P*-value	SALL4 expression		*p*-value
		High group	Low group		High group	Low group	
*Age(years)*							
≥60	32	17	15	0.82	18	14	0.494
<60	48	23	25		22	26	
Gender							
Male	50	24	26	0.818	23	27	0.489
Female	30	16	14		17	13	
Size(cm)							
≥3(cm)	42	14	28	0.003**	26	16	0.043*
<3(cm)	38	26	12		14	24	
Stage							
I/II	45	26	19	0.176	21	24	0.652
III/IV	35	14	21		19	16	
T grade							
T_1_ + T_2_	47	25	22	0.65	18	29	0.22
T_3_ + T_4_	33	15	18		22	11	
*Lymph node metastasis*							
N1–N3	48	18	30	0.012*	29	19	0.039*
N0	32	22	10		11	21	
Histology grade							
Well-moderately	34	19	15	0.498	13	21	0.113
Poorly-signet	46	21	25		27	19	

*p < 0.05 and **p < 0.01 Statistically significant difference.

### MiR-3622a-3p inhibited proliferation of CRC cells

**Fig. 2:**
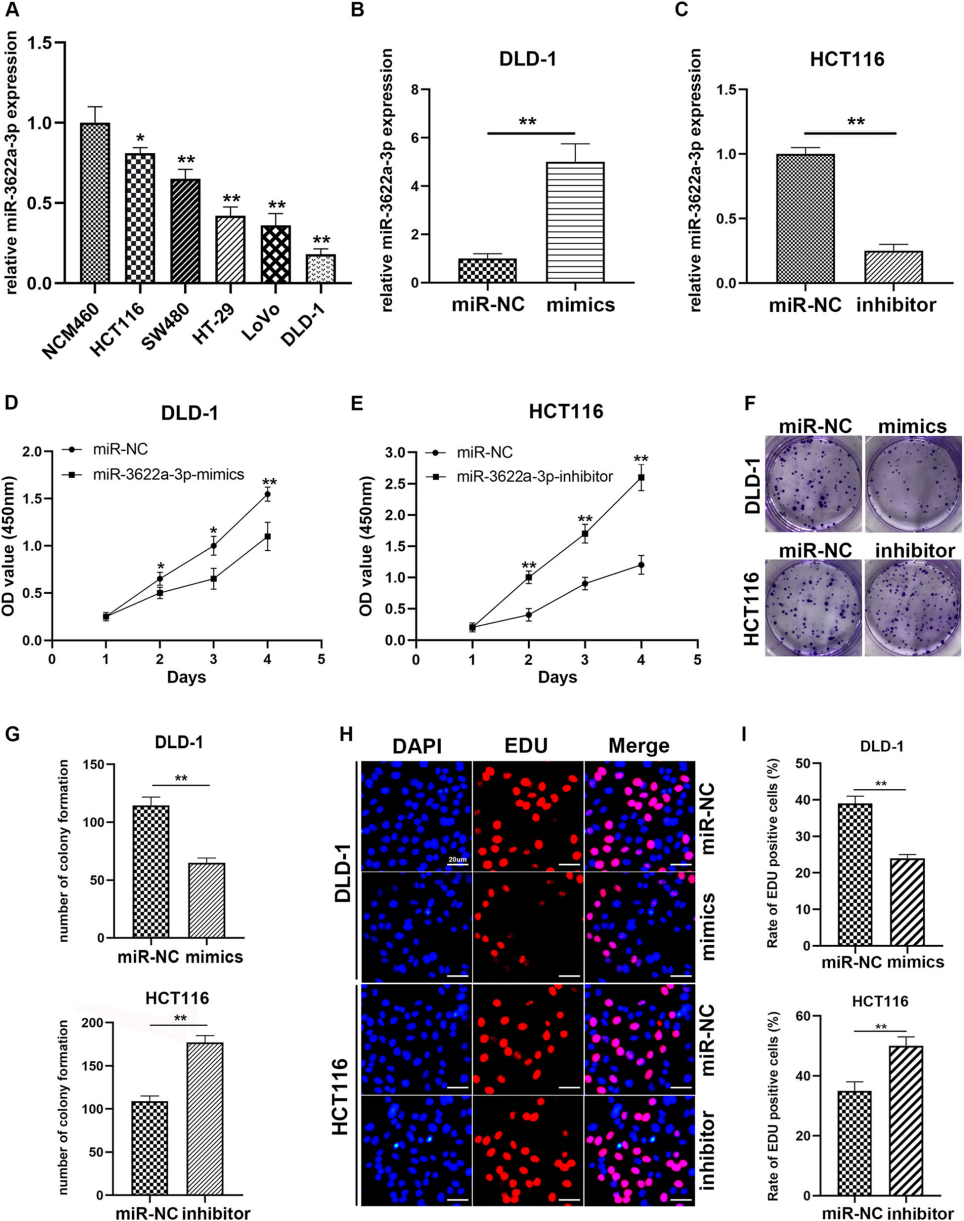
MiR-3622a-3p inhibited proliferation of CRC cells. **a** Expression level of miR-3622a-3p was detected in CRC cell lines and NCM460 cell line by qRT-PCR. **b** Expression of miR-3622a-3p was increased in DLD-1 by miR-3622a-3p mimics transfection. **c** MiR-3622a-3p expression level was reduced in HCT116 by miR-3622a-3p inhibitor transfection. **d**, **e** The effect of miR-3622a-3p on proliferation of CRC cells was evaluated by CCK-8 cell proliferation assay. **f**, **g** Colony forming ability of CRC cells was negatively corelated with miR-3622a-3p expression level. **h**, **i** The results of EDU assay suggested overexpression of miR-3622a-3p suppressed proliferation of CRC cells while knockdown of miR-3622a-3p promoted CRC cell proliferation. All data are from three independent experiments and are presented as the means ± SD (*p < 0.05, **p < 0.01).

We performed qRT-PCR to detect miR-3622a-3p expression in CRC cells. As shown in Fig. 2a, compared with human normal colon epithelial cell line NCM460, miR-3622a-3p was decreased in different CRC cell lines. DLD-1 and LoVo were selected to be transfected with miR-3622a-3p mimics while HCT116 and SW480 were transfected with miR-3622a-3p inhibitor. We performed qRT-PCR to determine the transfection efficiency and found miR-3622a-3p was remarkably overexpressed in DLD-1, LoVo and obviously reduced in HCT116, SW480 after transfection (Fig. 2b, c and Supplementary Fig. 2A, B). To explore the effect of miR-3622a-3p on proliferation of CRC cells, we performed CCK-8 cell proliferation assay. Overexpression of miR-3622a-3p suppressed DLD-1 and LoVo proliferation in contrast with control group while knockdown of miR-3622a-3p contributed to growth of HCT116 and SW480 (Fig. 2d, e and Supplementary Fig. 2C, D). The results of colony formation assay indicated that miR-3622a-3p expression level was negatively corelated with colony forming ability of CRC cells (Fig. 2f, g and Supplementary Fig. 2E, F). The EDU assay was also employed to evaluate cell proliferation. We found up-regulation of miR-3622a-3p promoted DNA synthesis of CRC cells while miR-3622a-3p knockdown had the opposite effect (Fig. 2h, i and Supplementary Fig. 2G, H).

### MiR-3622a-3p induced apoptosis and G0/G1 cell cycle arrest of CRC cells

**Fig. 3:**
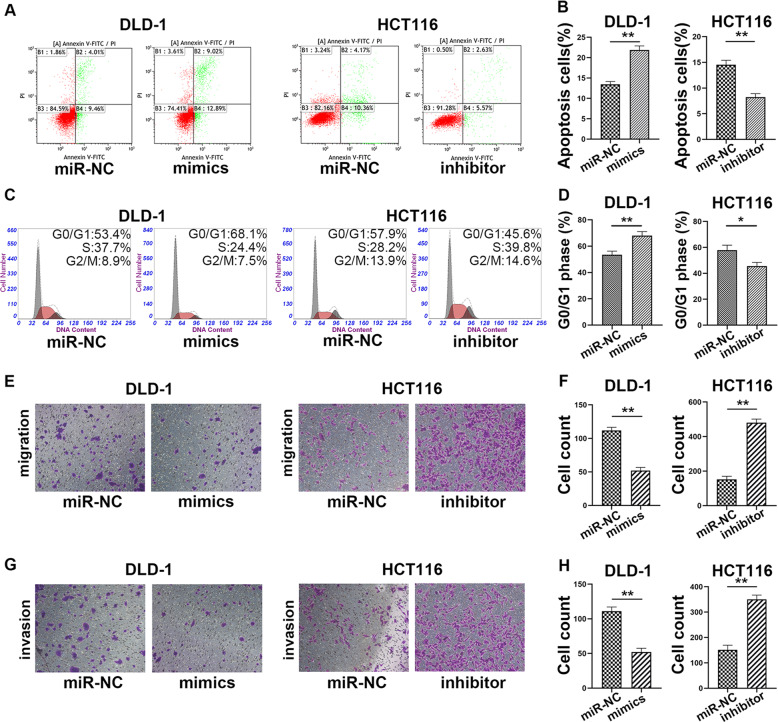
MiR-3622a-3p induced apoptosis and G0/G1 arrest while inhibited migration and invasion of CRC cells. **a**, **b** The effect of miR-3622a-3p on cell apoptosis was examined by flow cytometric analysis. **c**, **d** The effect of miR-3622a-3p on cell cycle was detected by flow cytometric analysis. **e**, **f** MiR-3622a-3p inhibited migration ability of CRC cells. **g**, **h** Transwell invasion assay was performed to assess the influence of miR-3622a-3p on CRC cell invasion ability. All data are from three independent experiments and are presented as the means ± SD (*p < 0.05, **p < 0.01).

To further study the effect of miR-3622a-3p on apoptosis and cell-cycle, flow cytometric analysis were employed. Apoptosis of DLD-1 and LoVo was induced by miR-3622a-3p overexpression while apoptosis of HCT116 and SW480 was reduced by knockdown of miR-3622a-3p (Fig. 3a, b and Supplementary Fig. 3A, B). DLD-1 and LoVo mimics group showed an obvious increase in the percentage of cells in G0/G1 phase while down-regulation of miR-3622a-3p achieved the opposite effect in HCT116 and SW480 (Fig. 3c, d and Supplementary Fig. 3C, D).

### MiR-3622a-3p suppressed migration and invasion ability of CRC cells

Since miR-3622a-3p expression level was negatively corelated with lymph node metastasis of CRC patients, we supposed that miR-3622a-3p might have an impact on migration and invasion ability of CRC cells. The migration abilities of DLD-1 and LoVo were impaired by miR-3622a-3p overexpression. HCT116 and SW480 transfected with miR-3622a-3p inhibitor exhibited a sharp increase in migration ability (Fig. 3e, f and Supplementary Fig. 3E, F). Judged from results of cell invasion assay, overexpression of miR-3622a-3p reduced invasion ability of CRC cells while knockdown of miR-3622a-3p acted the opposite way (Fig. 3g, h and Supplementary Fig. 3G, H).

### SALL4 was a direct target of miR-3622a-3p

**Fig. 4:**
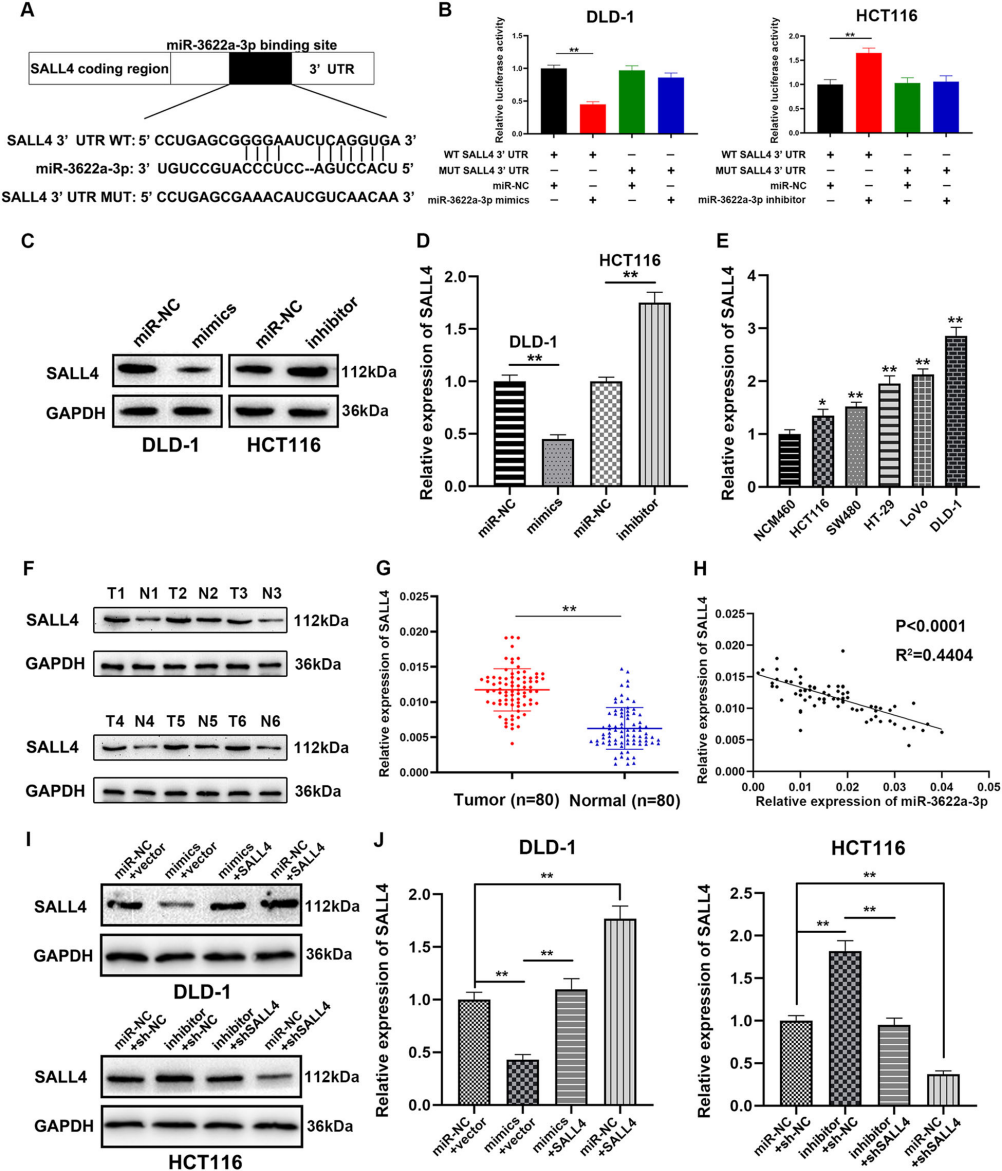
SALL4 was directly targeted by miR-3622a-3p and up-regulated in CRC. **a** The predicted binding of miR-3622a-3p at 3′UTR of SALL4 by online databases. **b** The luciferase reporter assay was performed to validate the interaction between miR-3622a-3p and SALL4. **c** SALL4 protein expression of stable transfected CRC cells was determined by western blot. **d** SALL4 was negatively regulated by miR-3622a-3p by qRT-PCR. **e** SALL4 was up-regulated in CRC cell lines compared with normal colon epithelial cell line by qRT-PCR. **f** SALL4 protein expression was detected by western blot in 6 pairs of CRC tissues and adjacent normal tissues. **g** qRT-PCR was performed on 80 pairs of CRC tissues and adjacent normal tissues for SALL4 detection. **h** SALL4 expression level was negatively corelated with miR-3622a-3p expression level in CRC specimens. **i** Western blot was used to verify protein expression of SALL4 in stable transfected CRC cells. **j** qRT-PCR was performed to determine the expression level of SALL4 in stable transfected CRC cells. All data are from three independent experiments and are presented as the means ± SD (*p < 0.05, **p < 0.01).

Online databases, including TargetScan (http://www.targetscan.org/) and miRDB (http://www.mirdb.org/) were used for prediction of target genes of miR-3622a-3p. The results of bioinformatics analysis suggested that SALL4, which has been reported to function as an oncogene in many types of cancers, could be a potential target of miR-3622a-3p (Fig. 4a). To further validate the interaction between miR-3622a-3p and SALL4, the dual luciferase reporter assay was adopted. As shown in Fig. 4b, co-transfection with pGL3-WT-SALL4 3′-UTR and miR-3622a-3p mimics resulted in reduced luciferase activity in DLD-1. In HCT116, co-transfection with pGL3-WT-SALL4 3′-UTR and miR-3622a-3p inhibitor showed an increased luciferase activity. However, luciferase activity was not affected by co-transfection with pGL3-MUT-SALL4 3′-UTR and miR-3622a-3p mimics or inhibitor. Overexpressed miR-3622a-3p generated obvious SALL4 enrichment in DLD-1 after Ago2 RIP while down-regulation of miR-3622a-3p had the opposite effect in HCT116 (Supplementary Fig. 4A, B). In addition, the results of pull-down assay also verified the interaction between miR-3622a-3p and SALL4 (Supplementary Fig. 4C, D). Western blot was employed to detect SALL4 protein expression in stable transfected CRC cells. Overexpression of miR-3622a-3p inhibited SALL4 expression whereas expression level of SALL4 was elevated by blocking of miR-3622a-3p (Fig. 4c). The similar results that SALL4 was negatively regulated by miR-3622a-3p were obtained by qRT-PCR (Fig. 4d). The results of qRT-PCR also suggested that SALL4 was up-regulated in different CRC cell lines compared with NCM460 (Fig. 4e). SALL4 protein expression was examined by western blot in 6 pairs of randomly chosen CRC tissues and adjacent normal tissues. The results indicated that SALL4 protein expression was remarkably increased in CRC tissues compared to their corresponding normal tissues (Fig. 4f). The results of qRT-PCR which was performed on 80 pairs of human CRC and normal samples revealed an up-regulated expression profile of SALL4 in CRC patients (Fig. 4g). The expression level of SALL4 decreased with increasing expression of miR-3622a-3p judged from results of linear correlation analysis (Fig. 4h). On the basis of above findings, SALL4 was validated to be a direct target of miR-3622a-3p.

### MiR-3622a-3p acted as a tumor suppressor by targeting SALL4

**Fig. 5:**
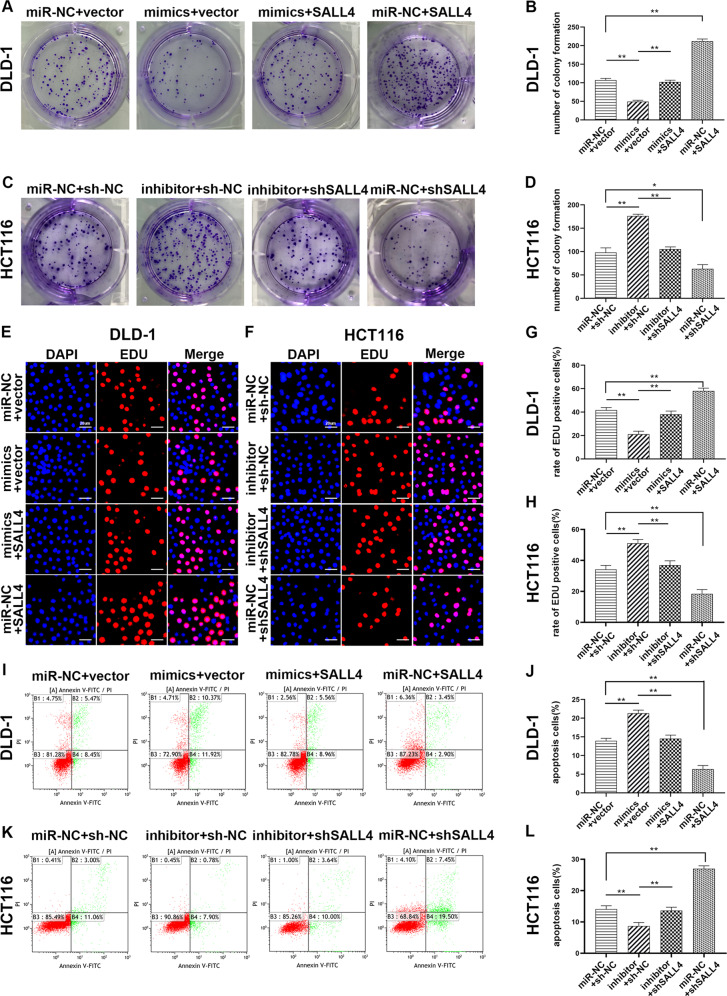
The effects of miR-3622a-3p and SALL4 on cell proliferation and apoptosis of CRC cells. **a**–**d** The effect of SALL4 on CRC cell proliferation was evaluated by colony formation assay. The rescue experiment for miR-3622a-3p overexpression was performed by ectopic expression of SALL4 in DLD-1. The rescue experiment for miR-3622a-3p silencing was performed by inhibition of SALL4 in HCT116. **e**–**h** The promoting role of SALL4 in CRC cell proliferation was further validated by the EDU assay and the rescue experiments were performed. **i**–**l** Flow cytometric analysis was employed to explore the impact of SALL4 on apoptosis of CRC cells and the rescue experiments were performed. All data are from three independent experiments and are presented as the means ± SD (*p < 0.05, **p < 0.01).

Since SALL4 was demonstrated to be directly targeted by miR-3622a-3p, we further explored the effect of SALL4 on CRC cells and whether functions of miR-3622a-3p could be mediated by regulation of SALL4. SALL4 expression levels in stable transfected CRC cells were confirmed by western blot and qRT-PCR (Fig. 4i, j). As shown in Fig. 5a, b, overexpression of SALL4 increased the number of colony formation of and reversed the influence of miR-3622a-3p on colony forming ability of DLD-1. Knockdown of SALL4 reduced the colony forming ability of HCT116 and counteract the promoting role of miR-3622a-3p down-regulation in colony formation (Fig. 5c, d). By adopting the EDU assay, overexpression of SALL4 was confirmed to contribute to cell proliferation of DLD-1 and up-regulated the cell proliferation which was suppressed by miR-3622a-3p (Fig. 5e, g). The contributing role of miR-3622a-3p knockdown in CRC cell proliferation was rescued by shSALL4 transfection (Fig. 5f, h). Flow cytometric analysis was employed to determine the impact of SALL4 on DLD-1 apoptosis and its ability to neutralize the effect of overexpression of miR-3622a-3p (Fig. 5i, j). ShSALL4 transfection increased apoptosis of HCT116 and could reverse the influence of down-regulation of miR-3622a-3p on HCT116 apoptosis (Fig. 5k, l). SALL4 overexpression decreased the percentage of cells in G0/G1 phase and counteracted the impact of miR-3622a-3p on cell cycle of DLD-1 (Supplementary Fig. 5A, B). The G0/G1 cell cycle arrest was induced by knockdown of SALL4 in HCT116 and the effect of miR-3622a-3p inhibitor transfection on cell cycle was rescued by knockdown of SALL4 (Supplementary Fig. 5C, D). The effects of SALL4 on migration and invasion abilities of CRC cells were investigated and the results indicated that SALL4 played a promotive role in cell migration and invasion. It was also confirmed that the effects of miR-3622a-3p on CRC cell migration and invasion were mediated by regulation of SALL4 (Supplementary Fig. 5E–L). Based on the results above, miR-3622a-3p inhibited malignant biological activities of CRC cells through targeting SALL4 directly.

### SALL4 abrogated the inhibitory effect of miR-3622a-3p on cancer stemness features of CRC cells

SALL4 is believed to promote stemness features in many types of cancers^16,34,35^.

**Fig. 6:**
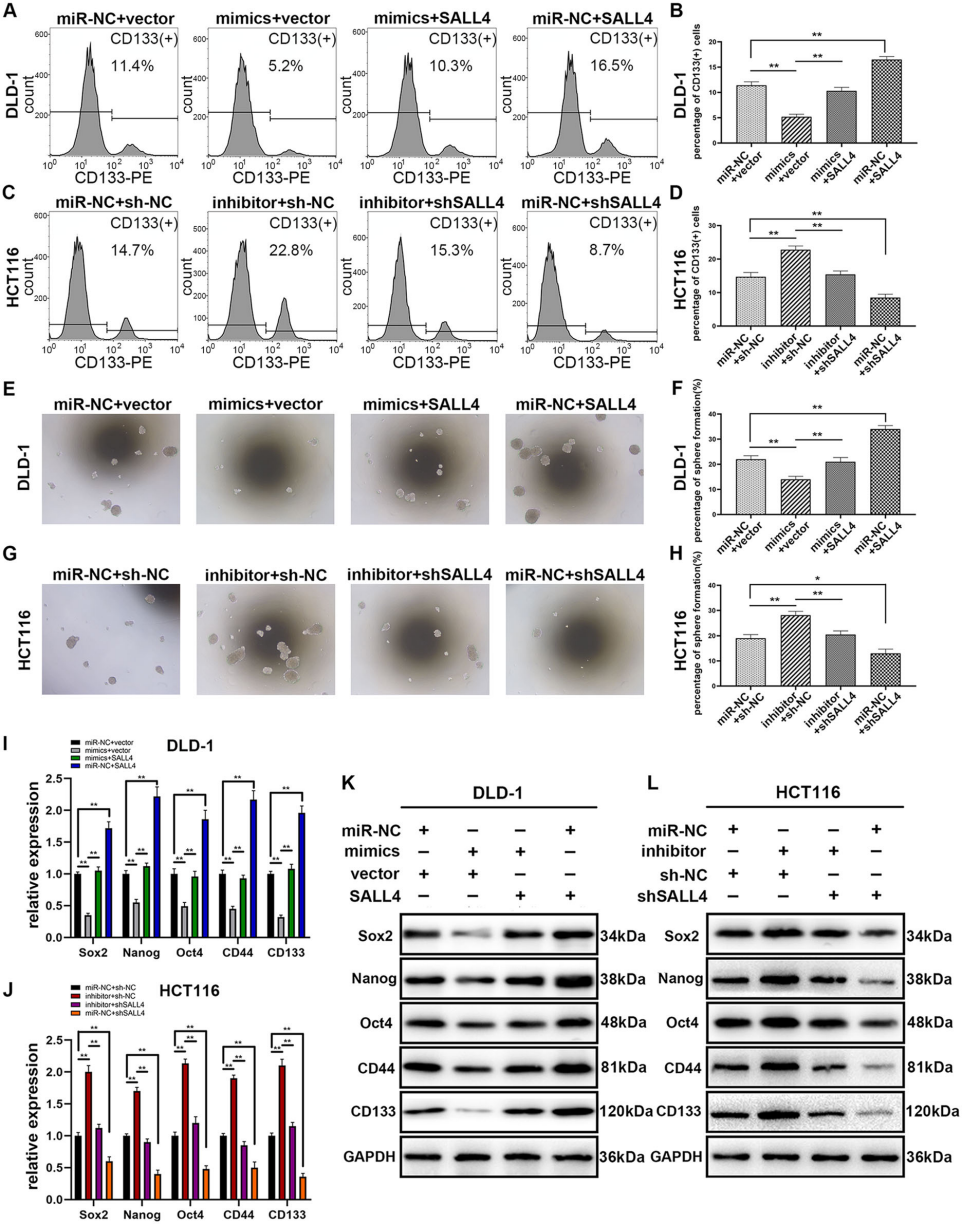
The effects of miR-3622a-3p and SALL4 on stemness features of CRC cells. **a**–**d** Flow cytometric analysis was used to examine the percentage of CD133(+) CRC cells. **e**–**h** Sphere formation assay was performed to investigate the stem-cell like properties of stable transfected CRC cells. **i**, **j** Expression levels of Sox2, Nanog, Oct4, CD44, and CD133 were determined by qRT-PCR in stable transfected CRC cells. **k**, **l** Protein levels of CSC-related biomarkers and pluripotency-related genes were detected by western blot in stable transfected CRC cells. All data are from three independent experiments and are presented as the means ± SD (*p < 0.05, **p < 0.01).

Thus we tried to figure out the effect of miR-3622a-3p on stemness features of CRC and whether the effect could be reversed by SALL4. It has been reported that CD133 could characterize cancer stem-like cells (CSCs) population in many types of tumors^36^, so flow cytometric analysis was used to detect CD133(+) CRC cells. As shown in Fig. 6a, b, overexpression of miR-3622a-3p decreased the percentage of CD133(+) cells in DLD-1 and the inhibitory effect was reversed by SALL4 overexpression. The promotive effect of miR-3622a-3p down-regulation on CD133(+) cell population was counteracted by shSALL4 transfection in HCT116 (Fig. 6c, d). It was observed in Fig. 6e, f that miR-3622a-3p suppressed sphere formation of DLD-1 and the effect was rescued by SALL4 overexpression. As shown in Fig. 6g, h, the contributing role of miR-3622a-3p knockdown in sphere formation was neutralized by down-regulation of SALL4. MiR-3622a-3p overexpression inhibited protein levels of CSC-related biomarkers and pluripotency-related genes in CRC cells while knockdown of miR-3622a-3p had the opposite effect. The results of qRT-PCR and western blot also suggested that the influences of overexpression or knockdown of miR-3622a-3p on CSC-related biomarkers and pluripotency-related genes could be reversed by SALL4 up-regulation or down-regulation respectively (Fig. 6i–l).

### MiR-3622a-3p was involved in inhibition of EMT and Wnt/beta-catenin signaling pathway through targeting SALL4

**Fig. 7:**
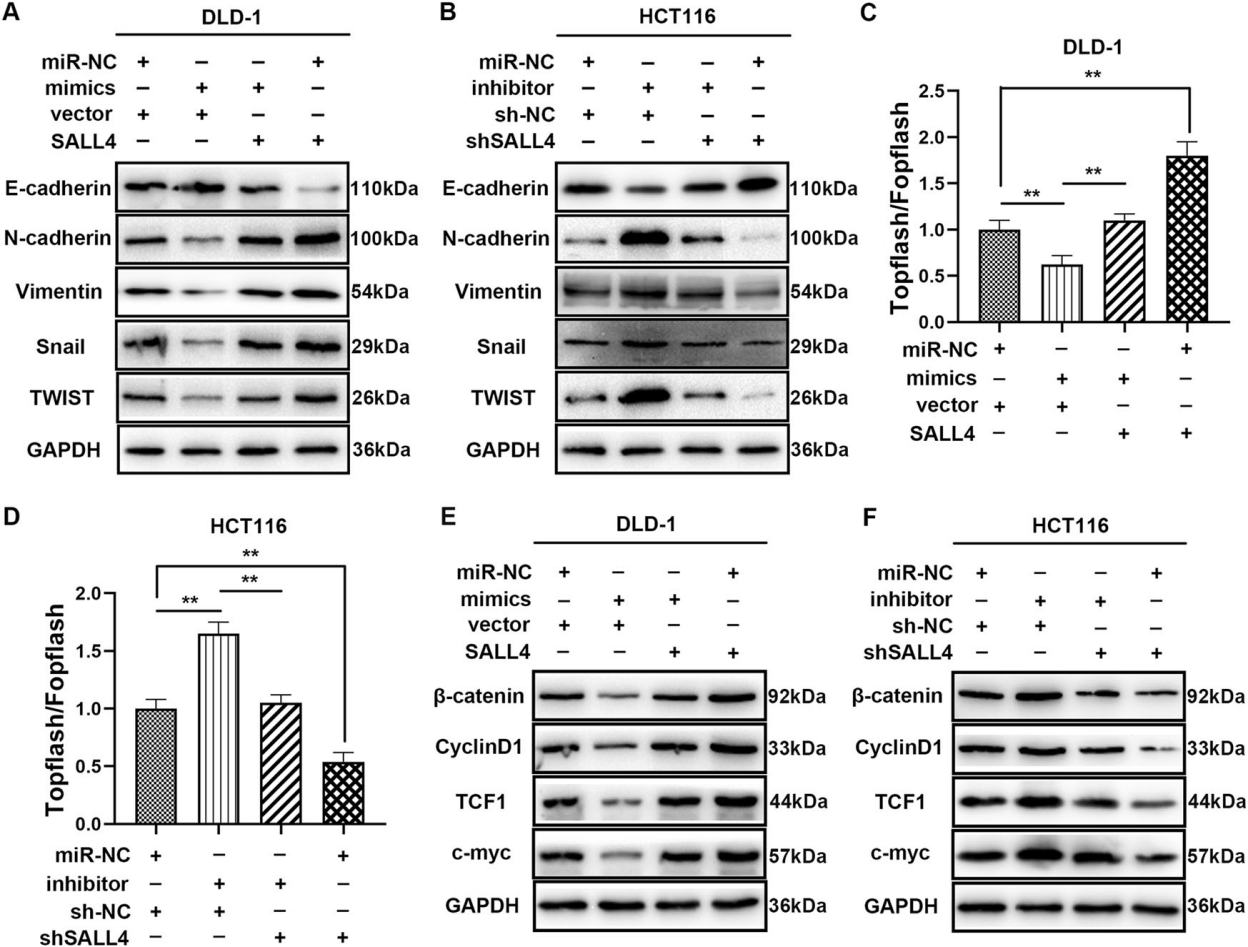
MiR-3622a-3p was involved in inhibition of EMT and Wnt/beta-catenin signaling pathway through targeting SALL4. **a**, **b** Protein levels of EMT markers were determined by western blot in stable transfected CRC cells. **c**, **d** The effects of miR-3622a-3p and SALL4 on Wnt/beta-catenin signaling pathway were explored by TOPflash/FOPflash luciferase reporter assay. **e**, **f** Expression levels of Wnt/beta-catenin signaling pathway related proteins were detected by western blot. All data are from three independent experiments and are presented as the means ± SD (*p < 0.05, **p < 0.01).

It has been reported that SALL4 contributes to EMT through Wnt/beta-catenin signaling pathway in different types of carcinomas^37,38^, so we evaluated the relationship between miR-3622a-3p, SALL4 and EMT. The EMT-related markers were examined by western blot and the results were shown in Fig. 7a, b. Overexpression of miR-3622a-3p suppressed EMT and the change could be mitigated by SALL4 up-regulation. Silencing of miR-3622a-3p positively regulated EMT and the effect was alleviated by shSALL4 transfection. To explore the effects of miR-3622a-3p and SALL4 on Wnt/beta-catenin signaling pathway, TOPflash/FOPflash luciferase reporter assay was employed. The results demonstrated that miR-3622a-3p inhibited Wnt/beta-catenin signaling pathway while SALL4 exerted the opposite effect and could counteract the influence of miR-3622a-3p (Fig. 7c). ShSALL4 transfection could inhibit the Wnt/beta-catenin signaling pathway which was activated by miR-3622a-3p inhibitor (Fig. 7d). Wnt/beta-catenin signaling related proteins were detected by western blot. As shown in Fig. 7e, f, miR-3622a-3p played an inhibitory role in Wnt/beta-catenin signaling pathway and the effect could be reversed by SALL4. To sum up, miR-3622a-3p reduced Wnt/beta-catenin signaling pathway-mediated EMT by targeting SALL4.

### MiR-3622a-3p inhibited growth and metastasis of CRC cells in vivo

MiR-3622a-3p was demonstrated to suppress CRC in vitro in the above experiments and we were interested in whether miR-3622a-3p could exert similar influences in vivo.

**Fig. 8:**
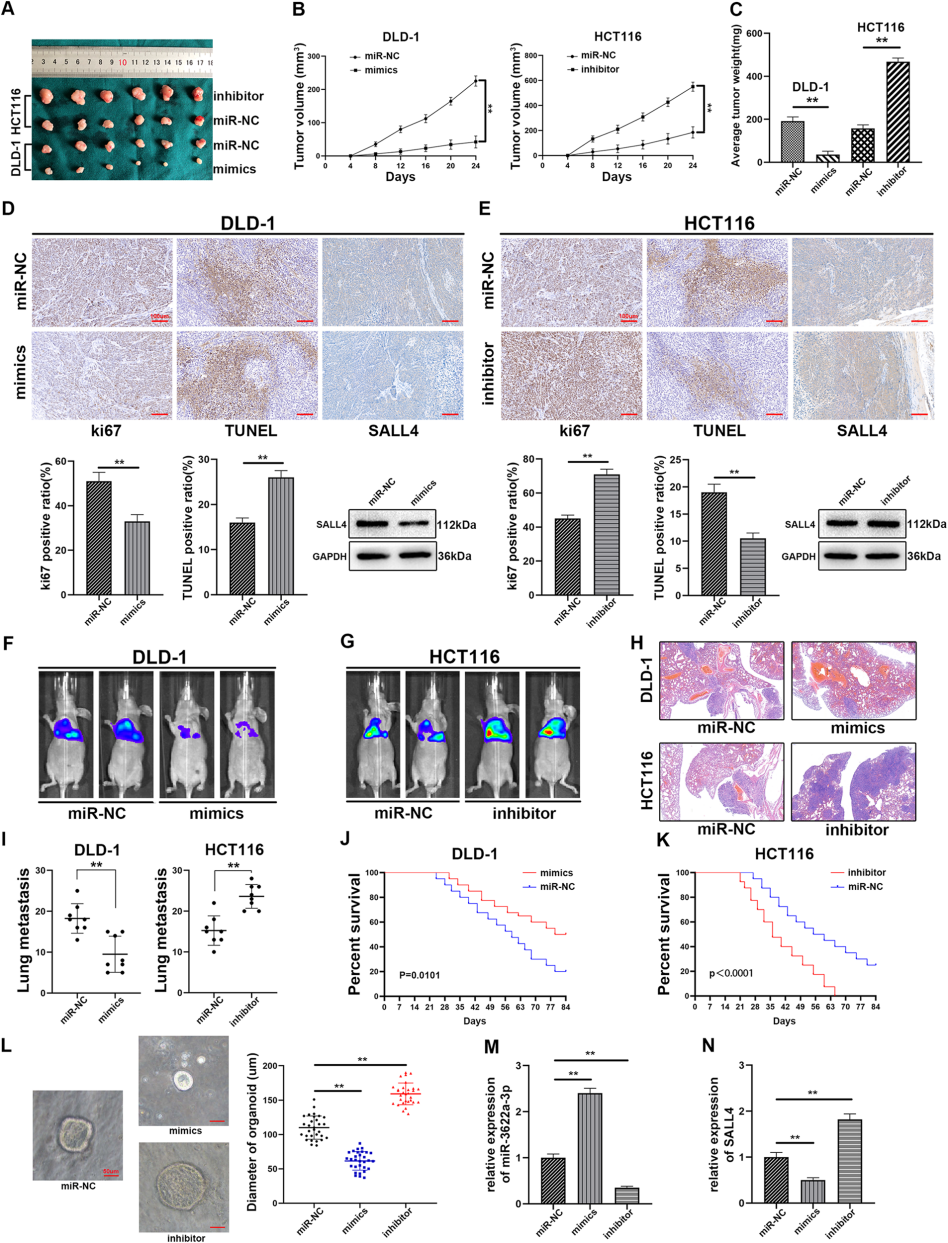
MiR-3622a-3p suppressed proliferation and metastasis of CRC cells in vivo. **a** Representative xenograft tumors derived from nude mice of different groups. **b** The growth curves of the xenograft tumors were drawn according to tumor volumes which were measured every 4 days. **c** The weight of the harvested tumors was recorded on day 24. **d** The results of ki67 staining and TUNEL assay revealed that overexpression of miR-3622a-3p inhibited proliferation and promoted apoptosis of DLD-1 xenograft tumors. SALL4 protein expression level was confirmed by IHC and western blot to be down-regulated by miR-3622a-3p overexpression. **e** Knockdown of miR-3622a-3p promoted proliferation and inhibited apoptosis of HCT116 xenograft tumors. SALL4 protein expression level was elevated by miR-3622a-3p knockdown. **f**, **g** Overexpression of miR-3622a-3p suppressed lung metastasis of CRC cells in vivo while silencing of miR-3622a-3p exerted the opposite influence. **h**, **i** Hematoxylin and eosin staining were performed on the harvested lungs from nude mice and the number of metastatic foci was counted. **j**, **k** High miR-3622a-3p expression level was a favorable factor for OS of nude mice while low miR-3622a-3p expression level was a risk factor. **l** The effects of miR-3622a-3p overexpression and knockdown on growth of CRC organoids. **m** MiR-3622a-3p expression in CRC organoids was detected by qRT-PCR following transfection with miR-3622a-3p mimics or inhibitor lentivirus. **n** The expression level of SALL4 in CRC organoids was determined by qRT-PCR. All data are from three independent experiments and are presented as the means ± SD (*p < 0.05, **p < 0.01).

Stable transfected CRC cells (1 × 10^6^) were injected into the flanks of nude mice to study the impact of miR-3622a-3p on tumor growth in vivo. The nude mice were sacrificed on day 24 and the tumors were harvested. It was observed that silencing of miR-3622a-3p facilitated tumor growth while overexpression of miR-3622a-3p exerted an opposite influence (Fig. 8a–c). We performed ki67 staining, TUNEL assay, IHC and western blot on the harvested tumors. MiR-3622a-3p inhibited proliferation and promoted apoptosis of DLD-1 in vivo. SALL4 protein expression was also down-regulated by miR-3622a-3p overexpression (Fig. 8d). As shown in Fig. 8e, silencing of miR-3622a-3p facilitated proliferation and inhibited apoptosis of HCT116. MiR-3622a-3p silencing also increased SALL4 protein expression. The tumor metastasis model was established by injecting 1 × 10^6^ stable transfected CRC cells into lateral tail veins of nude mice. As shown in Fig. 8f, g, lung metastases of nude mice were negatively associated with miR-3622a-3p expression. Eight nude mice of each group were sacrificed and hematoxylin and eosin staining were performed on the harvested lungs (Fig. 8h). The number of metastatic foci were also counted (Fig. 8i). The rest of nude mice in each group were kept for 12 weeks for survival analysis. As shown in Fig. 8j and Fig. 8k, nude mice in miR-3622a-3p overexpression and knockdown group exhibited longer and shorter overall survival (OS) compared with control group respectively.

### MiR-3622a-3p negatively regulated the growth of CRC organoids

Patient-derived tumor organoid model has emerged as an effective tool for biomedical research. The organoid model allows culture of stem cells in a 3D circumstance^39^. The model facilitates investigation into various diseases, including different kinds of cancers^40^. MiR-3622a-3p overexpression contributed to growth of CRC organoids, whereas silencing of miR-3622a-3p resulted in the opposite trend (Fig. 8l). The transfection efficiency was determined by qRT-PCR (Fig. 8m). The expression level of SALL4 was also detected by qRT-PCR in the organoids which were stably transfected with miR-3622a-3p mimics or inhibitor lentivirus (Fig. 8n).

## Discussion

The incidence and lethality of CRC has been reported to be the third and second respectively among all the malignancies globally^41^. The molecular mechanism underlying CRC which is vital for better treatment strategy has not been clarified yet. Aberrant expression of miRNAs has been acknowledged to be involved in initiation and development of CRC. For example, miR-4260 plays a promotive role in CRC by targeting MCC and SMAD4^42^. MiR-494 activates Wnt/beta-catenin signaling pathway through APC mRNA degradation in CRC^43^. MiR-99b-5p inhibits liver metastasis of CRC through targeting mTOR^44^. By analyzing the data from TCGA database, we found that miR-3622a-3p was remarkably down-regulated in CRC tissues compared with normal ones. MiR-3622a-3p has been reported to be involved in bladder cancer and prostate cancer^9,10^, but its role in CRC remains unknown. Then miR-3622a-3p expression was confirmed to decrease in CRC by performing qRT-PCR on 80 pairs of CRC tissues and adjacent normal tissues. We also analyzed the overall survival of the CRC patients recruited to the study and found miR-3622a-3p was a positive factor for diagnosis of the patients. According to the clinicopathological features of the CRC patients, miR-3622a-3p was negatively corelated with CRC tumor size and lymph node metastasis. Based on the above findings, we hypothesized that miR-3622a-3p might act as a tumor suppressor in CRC and further research needed to be done.

To explore the effects of miR-3622a-3p on CRC cells, several biological function assays were conducted. By CCK-8 proliferation assay, colony formation assay and EDU assay, miR-3622a-3p was validated to inhibit proliferation of CRC cells. The results of flow cytometric analysis revealed that miR-3622a-3p could induce apoptosis and G0/G1 cell cycle arrest in CRC cells. Considering miR-3622a-3p was negatively corelated with lymph node metastasis, Transwell migration and invasion assays were employed and we observed the migration and invasion abilities of CRC cells were reduced by miR-3622a-3p. MiRNAs exert functions by degrading mRNAs of targeted genes, so two online databases, including TargetScan and miRDB were used for prediction of miR-3622a-3p downstream targeted genes. SALL4, which has been demonstrated to serve as a potential diagnostic and prognostic biomarker for CRC^45^, was calculated to be a candidate. Dual luciferase assay, RIP assay and pull-down assay were adopted to confirm the interaction between miR-3622a-3p and SALL4. Expression level of SALL4 was determined by qRT-PCR and western blot to be negatively corelated with miR-3622a-3p expression level. The results of qRT-PCR and western blot also revealed SALL4 was up-regulated in CRC cells and tissues. The expression level of SALL4 was observed to decrease with increasing of miR-3622a-3p in CRC tissues by linear correlation analysis. Rescue experiments were conducted to find out whether functions of miR-3622a-3p could be mediated by regulation of SALL4. Through biological function assays, overexpression of SALL4 could reverse the effect of miR-3622a-3p on CRC cell proliferation, apoptosis, cell cycle, migration and invasion. The influence of miR-3622a-3p knockdown could also be reversed by silencing of SALL4.

SALL4 is not only essential for pluripotency and self-renewal of ESCs, but also important for regulation of CSCs maintenance^46^, hence we studied the effects of miR-3622a-3p and SALL4 on stemness features of CRC. MiR-3622a-3p decreased the percentage of CD133(+) CRC cells and reduced the sphere forming ability of CRC cells.

Expression levels of CSC-related biomarkers and pluripotency-related genes were also down-regulated by miR-3622a-3p. However, SALL4 contributed to stemness features of CRC and could counteract the inhibitory effect of miR-3622a-3p on stemness features. Activation of EMT is believed to enhance migration and invasion abilities of tumor cells^47^. Now that miR-3622a-3p was demonstrated to regulate migration and invasion abilities of CRC cells in our study and SALL4 has been reported to induce EMT by Wnt/beta-catenin signaling pathway^37,38^, the effects of miR-3622a-3p on EMT and Wnt/beta-catenin signaling pathway were explored. As expected, miR-3622a-3p resulted in inhibition of EMT and inactivation of Wnt/beta-catenin signaling pathway.

Xenograft tumor model and tumor metastasis model were established using nude mice for further research on the impacts of miR-3622a-3p on CRC cells in vivo. We found that miR-3622a-3p could also inhibit proliferation and promote apoptosis of CRC cells and regulate SALL4 expression in vivo. In addition, lung metastasis of CRC cells in nude mice was restrained by miR-3622a-3p. The organoid model is widely applied for the research on the stem-cell characteristics of human CSCs^48^. Wnt/beta-catenin signaling pathway activation is vital for the process of organoid development and differentiation^49^. The suppressive effects of miR-3622a-3p on stemness features and Wnt/beta-catenin signaling pathway might account for inhibition of organoid growth after being transfected with miR-3622a-3p mimics.

There were still several limitations to our study. Circulating miRNAs have been reported to function as biomarkers for early detection of CRC^50^. However, the expression level of miR-3622a-3p was only detected in tissue samples rather than plasma specimens. Wnt/beta-catenin signaling pathway was confirmed to be inactivated by miR-3622a-3p, but we cannot rule out the possibility that miR-3622a-3p might regulate other signaling pathways in CRC. The CRC organoid model has already been used to study chemoresistance of CRC^51^. The application of the organoid model in our study was only limited to laboratory research. We will try to construct more human CRC organoids and develop individual treatment plans for CRC patients based on the responses of organoids to chemotherapeutic drugs.

Our data revealed that miR-3622a-3p was aberrantly down-regulated in CRC and inhibited progression and metastasis of CRC cells in vitro and in vivo. High expression level of miR-3622a-3p was an advantage for prognosis of CRC patients. MiR-3622a-3p exerted anticancer influences by decreasing SALL4-mediated stemness features and inactivating EMT and Wnt/beta-catenin signaling pathway in CRC. Our research on miR-3622a-3p might help understanding the molecular mechanisms underlying CRC and provide new insights into therapeutic strategies for CRC.

## Materials and methods

### Human tissue specimens

The 80 pairs of CRC tissue samples and adjacent normal tissue samples were obtained from Department of General Surgery, the First Affiliated Hospital of Nanjing Medical University. The patients recruited to our study did not receive preoperative chemotherapy and radiotherapy. All the patients signed written informed consents before the study. The research was approved by theInstitutionalEthical Board of the First Affiliated Hospital of Nanjing Medical University. The clinical stages of the CRC patients were determined according to the International Union Against Cancer (UICC) on Tumor-Node-Metastasis (TNM) staging system (7th edition). The collected tissues samples were stored in liquid nitrogen before use.

### Cell lines and cell culture

The five CRC cell lines, including HCT116, SW480, HT-29, LoVo and DLD-1 were purchased from the Cell Bank of Type Culture Collection of the Chinese Academy of Sciences (Shanghai, China). Human normal colon epithelial cell line NCM460 was obtained from American Type Culture Collection (ATCC, USA). Cell lines were authenticated using Short Tandem Repeat (STR) analysis and tested for mycoplasma contamination. All of the cell lines were cultured in DMEM medium (Wisent, Canada), containing 10% fetal bovine serum (FBS, Wisent, Canada), 100 U/ml penicillin and streptomycin (15140148, Invitrogen, USA) in a moist incubator with 5% CO_2_ at 37 °C.

### Cell transfection

MiR-3622a-3p mimics lentivirus, miR-3622a-3p inhibitor lentivirus, the lentiviral vector containing SALL4 DNA sequencing (LV-SALL4) and the lentiviral vector containing SALL4 shRNA sequence (sh-SALL4) were purchased from GenePharma (Shanghai, China). We used puromycin (Sigma, Aldrich) to screen the transfected CRC cells to establish stable transfected cell lines according to the manufacturer’s protocol.

### RNA extraction and quantitative real-time polymerase chain reaction (qRT-PCR)

Total RNAs were extracted with Trizol Reagent (15596018, Invitrogen, USA) from CRC tissues and cell lines. PrimeScript RT Reagent (RR047A, Takara, Japan) and New Poly(A) Tailing Kit (ThermoFisher, China) were used for mRNA and miRNA reverse transcription respectively into cDNA followingthemanufacturer’s instructions. We carried out qRT-PCR with a 7500 Realtime PCR System (Applied Biosystems, USA) and SYBR Green Master Mix (4913914001, Roche, USA). Relative expression of miR-3622a-3p was normalized to snRNA U6. Beta-actin was taken as internal control for pri/pre-miR-3622a-3p, SALL4, Sox2, Nanog, Oct4, CD44 and CD133 detection. The 2^−ΔΔCT^ analysis method was used to calculate the relative expression of miR-3622a-3p and SALL4. The primers for qRT-PCR were listed below: has-miR-3622a-3p forward, 5′-TCACCTGACCTCCCATGCCTGT-3′; Universal, 5′-GCGAGCACA GAATTAATACGAC-3′; U6 forward, 5′-CTCGCTTCGGCAGCACA-3’; U6 reverse, 5′-AACGCTTCACGAATTTGCGT-3′; pri-miR-3622a-3p forward, 5′-TCGTGAGCTGCTTGATGACTGAT-3′; pri-miR-3622a-3p reverse, 5′-AGGAAGCCCAGGAAACCCTTTG-3′; pre-miR-3622a-3p forward, 5′-ACCTGACCTCCCATGCCT-3′; pre-miR-3622a-3p reverse, 5′-TATGCTTGTTCTCGTCTCTGTGTC-3′; SALL4 forward, 5′-TCGATGGCCAACTTCCTTC-3′; SALL4 reverse, 5′-GAGCGGACTCACACTGGAGA-3′; beta-actin forward, 5′-GCATCGTCACCAACTGGGAC-3′; beta-actin reverse, 5′-ACCTGGCCGTCAGGCAGCTC-3′; Sox2 forward, 5′-ACACCAATCCCATCCACACT-3′; Sox2 reverse, 5′-GCAAACTTCCTGCAAAGCTC-3′; Nanog forward, 5′-CCTGATTCTTCCACCAGTCC-3′; Nanog reverse, 5′-TGCTATTCTTCGGCCAGTTG-3′; Oct4 forward, 5′-TTGAGGCTCTGCAGCTTAG-3′; Oct4 reverse, 5′-GCCGGTTACAGAACCACAC-3′; CD44 forward, 5′-TCACAGGTGGAAGAAGAGAC-3′; CD44 reverse, 5′-CATTGCCACTGTTGATCACT-3′; CD133 forward, 5′-CTGGGGCTGCTGTTTATTATTCTG-3′; CD133 reverse, 5′-ACGCCTTGTCCTTGGTAGTGTTG-3′.

### DNA methylation analysis

Genomic DNA was extracted from NCM460, DLD-1, LoVo cell lines and modified with bisulfite. The CpG islands were amplified by PCR and the products were separated by agarose gel electrophoresis and cloned into the pUC18 T-vector (Sango, Shanghai). DNA sequencing on 10 clones was performed after bacterial amplification of the cloned PCR fragments.

### Cell proliferation assay

To assess proliferation of stable transfected cells, we used a Cell Counting Kit-8 (CK04, Dojindo, Kumamoto, Japan). In all, 2 × 10^3^ cells were seeded into each well of a 96-well plate. The cells in each well were incubated with 10 μl CCK8 reagent for 2 h at 37 °C. Absorbance at 450 nm was measured by a microplate reader at the same time point for 5 days.

### 5-Ethynyl-2′-deoxyuridine (EDU) assay

DNA synthesis of stable transfected CRC cells was measured with an EDU assay kit (C00052, RiboBio, China). The cells were seeded at a density of 2 × 10^4^ cells per well into a 24-well plate and cultured in DMEM containing 10% FBS for 24 h. After incubation with 50 uM EDU reagent at 37 °C for 2 h, the cells were fixed and permeabilized with 4% formaldehyde and 0.5% TritonX-100 respectively at room temperature (RT). Then we added 1 × Apollo R reaction cocktail (400 μl) to each well. After 30 min, 400 μl Hoechest33342 was added to stain the nuclei of stable transfected cells. Red and blue signals were observed and taken by a Nikon microscope (Nikon, Japan).

### Colony formation assay

The stable transfected cells were seeded into a six-well plate (500 cells/well) and cultured in DMEM (10% FBS) for 15 days. After being fixed with 75% ethyl alcohol, the colonies were stained with crystal violet (Beyotime, China). We washed the colonies with phosphate buffered solution (PBS) for three times and counted the number of colonies.

### Flow cytometric analysis

The stable transfected cells were digested with trypsin and then collected. After being washed with PBS twice and fixed with 75% ethyl alcohol, the cells were stored at −20 °C overnight. The cells were then washed with PBS, incubated with RNase and stained with a Cell Cycle Staining Kit (CCS012, Multi Sciences, China) for 15 min in the dark. The cell-cycle was analyzed with a FACScan flow cytometer (BD, USA). To evaluate apoptosis of stable transfected cells, the cells were collected and stained with a PE Annexin V Apoptosis Detection Kit I (559763, BD) based on the manufacturer’s protocol. The ratio of apoptotic cells was determined by a FACScan flow cytometer (BD, USA). The collected stable transfected cells were incubated with PE-conjugated CD133 antibody (372804, Biolegend, USA) at RT for 90 min. Then the cells were placed on ice for 10 min and washed with precooled PBS before flow cytometric analysis for CD133(+) cells detection.

### Transwell migration and invasion assays

To assess the migration ability of stable transfected cells, we used a Transwell plate (Corning, USA). In all, 2 × 10^4^ stable transfected cells were seeded into the upper chamber and cultured in 200 ul DMEM without FBS. In all, 500 μl DMEM medium containing 10% FBS which acted as chemoattractant was added to the lower chamber. The plate was incubated at 37 °C for 24 h. Part of the cell migrated to the underside of the membrane. The cells which did not penetrate the membrane were removed and the cells on the lower surface of the membrane were fixed and stained with 75% alcohol and crystal violet respectively. The stained cells were counted with a microscope. To perform invasion assay, 100 ul Matrigel (354248, Corning, USA) was coated on the upper side of the membrane before cell plating. The remaining steps were similar to those in migration assay.

### Dual-luciferase reporter assay

The 3’-UTR sequences of SALL4 containing wild-type (WT) or mutated (MUT) miR-3622a-3p binding site were designed and synthesized by GeneScript (Nanjing, China). The sequences were cloned into a a pGL-3 luciferase reporter vector (Promega, USA). DLD-1 was co-transfected with miR-3622a-3p mimics or miR-NC and pGL3-WT-SALL4 or pGL3-MUT-SALL4. HCT116 was co-transfected with miR-3622a-3p inhibitor or miR-NC and pGL3-WT-SALL4 or pGL3-MUT-SALL4. The luciferase activity was determined with a Dual Luciferase Reporter Assay System (Promega, USA). The ratio of firefly luciferase to renilla luciferase was defined as the relative luciferase activity.

### RNA immunoprecipitation assay

RNA immunoprecipitation (RIP) assay was carried out with a Magna RNA immunoprecipitation kit (Millipore, USA). Stable transfected CRC cells were lysed with RIP buffer. The cell lysis was incubated with magnetic beads conjugated with anti-Ago2 antibody (03–110, Millipore) or IgG antibody at 4 °C. Finally, the immunoprecipitated RNA was extracted and followed by qRT-PCR.

### Pull-down assay

Biotinylated-miR-3622a-3p (Bio-miR-3622a-3p) and biotinylated-miR-NC (Bio-NC) were purchased from GenePharma (Shanghai, China). DLD-1 and HCT116 were transfected with the constructs. In all, 48 h after transfection, the CRC cells were harvested and lysed. The cell lysis was incubated with streptavidin-coated magnetic beads (Invitrogen, CA) for 10 min. After being washed with PBS, the biotin-coupled RNA complex was pulled down and followed by qRT-PCR.

### Western blot analysis

RIPA lysis buffer (P0013C, Beyotime, China) was used to extract total proteins from paired CRC tumors and adjacent normal tissues and CRC cells. The proteins were separated by sodium dodecyl sulfate polyacrylamide gel electrophoresis (SDS-PAGE) and then transferred to a polyvinylidene fluoride (PVDF) membrane (Millipore, USA). After being blocked in 5% non-fat milk at RT for 2 h, the membranes were incubated with primary antibodies at 4 °C all through the night. The membranes were washed with TBST buffer for three times and incubated with secondary antibodies at RT for 2 h the next day. Finally, we washed the membranes for three times using TBST buffer and the proteins on the membranes were visualized by an enhanced chemiluminescence (ECL) detection system (Millipore, USA). The primary antibodies included: anti-SALL4 (ab57577), anti-GAPDH (ab8245), anti-Sox2 (ab79351), anti-Nanog (ab21624), anti-Oct4 (ab19857), anti-CD44 (ab157107), anti-CD133 (ab216323), anti-E-cadherin (ab1416), anti-N-cadherin (ab76057), anti-Vimentin (ab92547), anti-Snail (ab53519), anti-TWIST (ab50581), anti-beta-catenin (ab32572) and anti-Cyclin D1 (ab16663) from Abcam (Cambridge, UK), anti-TCF1 (#2203) and anti-c-myc (#5605) from Cell Signaling Technology (Boston, MA, USA). The secondary antibodies used in our study, including anti-rabbit IgG-HRP (ab6721) and anti-mouse IgG-HPR (ab6789) antibodies, were purchased from Abcam.

### Sphere formation assay

Stable transfected cells were seeded into a six-well ultra‐low attachment surface plate (Corning, USA) with 5 × 10^3^ cells per well. The cells were cultured in serum-free DMEM/F12 (Gibco, Australia). The culture medium was supplemented with B27 (Invitrogen, USA), N2 (Invitrogen, USA), EGF (Invitrogen, USA) and basic FGF (Invitrogen, USA). The spheres were observed and photographed 10 days later.

### TOPflash/FOPflash luciferase reporter assay

The Topflash/FOPflash reporter plasmids were obtained from Upstate Biotechnology (NY, USA). The cells were transfected with the plasmids using Lipofectamine 3000 (L3000015, Invitrogen, USA). The luciferase activity was detected by a Dual Luciferase Reporter Assay System (Promega, USA). The results were shown as normalized TOPFlash/FOPFlash values.

### Animal experiment

The BALB/c nude mice (aged 5 weeks) used in our study to build tumor xenograft model and tumor metastasis model were purchased from Animal Center of Nanjing Medical University (NJMU). The animal experiments were approved by NJMU Animal Ethics Committee. In all, 1 × 10^6^ CRC cells stably transfected with miR-3622a-3p mimics or inhibitor were injected into the flanks of nude mice (6 mice/group). The volume of the tumors was measured every 4 days with a vernier caliper following calculation formula: volume = (width^2^ × length)/2. The nude mice were sacrificed on day 24. For in vivo metastasis assay, 1 × 10^6^ stable transfected CRC cells suspended in 100 ul PBS were injected into lateral tail veins of nude mice. After 4 weeks, the distant metastases of 8 nude mice in each group were visualized with an IVIS Imaging system (Caliper life Sciences, USA). The remaining of the nude mice were kept to analyze the effect of miR-3622a-3p on survival of nude mice with 12 weeks as cutoff. All animal experiments were performed in accord with the National Institutes of Health Guide for the Care and Use of Laboratory Animals. Standard of blinding and randomization was complied with in this study.

### Immunochemical staining

The subcutaneous tumors of nude mice were obtained and fixed in 4% formaldehyde. Then they were embedded in paraffin and cut into 4 μm thick sections. The sections were incubated with primary antibodies, such as anti-ki67 (ab156956) and anti-SALL4 (ab57577) overnight at 4 °C. After being washed with PBS for three times, the sections were incubated with HRP-polymer-conjugated secondary antibody at RT for 1 h. We used 3,3′-Diaminobenzidine (DAB) solution to stain the sections for 3 min and hematoxylin to counterstain nuclei. The percentage of positive cells was determined based on three random fields of the sections.

### TUNEL assay

We used a TUNEL apoptosis detection kit (C1091, Beyotime, China) for this assay according to manufacturer’s protocol. The subcutaneous tumor sections were rehydrated in the ethanol and fixed in 4% formaldehyde. The sections were then incubated with proteinase K at RT for 20 min and 3% hydrogen peroxide was used to inactivate endogenous peroxidases. Working solution and chromogenic agent were prepared following manufacturer’s instructions. Hematoxylin was used to stain nuclei of the cells.

The percentage of apoptotic cells in randomly selected fields was determined with a microscope (Nikon, Japan).

### Hematoxylin and eosin staining

The lungs of the nude mice were fixed in 4% formaldehyde and embedded in paraffin. The sections prepared from paraffin mass was incubated with hematoxylin for 3 min and washed with deionized water. Then we used eosin Y solution to dye the sections and 95% alcohol followed by absolute ethanol to dehydrate the specimens. Eventually, xylene was adopted for alcohol extraction and neutral balsam was used to seal the sections.

### Establishment of CRC organoid model

The CRC organoid model was constructed based on the protocols published previously^39^. The organoids were transfected with miR-3622a-3p mimics or inhibitor lentivirus. After 12 days of culture, the diameters of the organoids were measured. Then the organoids were collected from Matrigel for miR-3622a-3p and SALL4 detection with qRT-PCR.

### Statistical analysis

All the statistical analyses adopted in the study were performed with Statistical Product and Service Solutions (SPSS) 20.0 software. The data was shown as mean ± standard deviation (SD). All the experiments were carried out at least three times. Linear correlation analysis was performed to analyze the correlation between miR-3622a-3p expression and SALL4 expression. χ2 test was used to determine the relationship between miR-3622a-3p expression level and the CRC patients’ clinicopathological features. The Caplan-Meier method was adopted in survival analysis. Two-tailed Student’s *t*-test and one-way analysis of variance (ANOVA) were performed to analyze the data obtained from experiments. *P* < 0.05 (*) and *P* < 0.01 (**) were considered statistically significant.

## Supplementary information

Supplementary Figure 1

Supplementary Figure 2

Supplementary Figure 3

Supplementary Figure 4

Supplementary Figure 5

Supplementary Figure legend
