# Supplementary figures and images for: MiR-3622a-3p acts as a tumor suppressor in colorectal cancer by reducing stemness features and EMT through targeting spalt-like transcription factor 4
Source: Cell Death Dis. 2020 Jul 27;11(7):592. doi: 10.1038/s41419-020-02789-z (PMC7385142; doi:10.1038/s41419-020-02789-z)

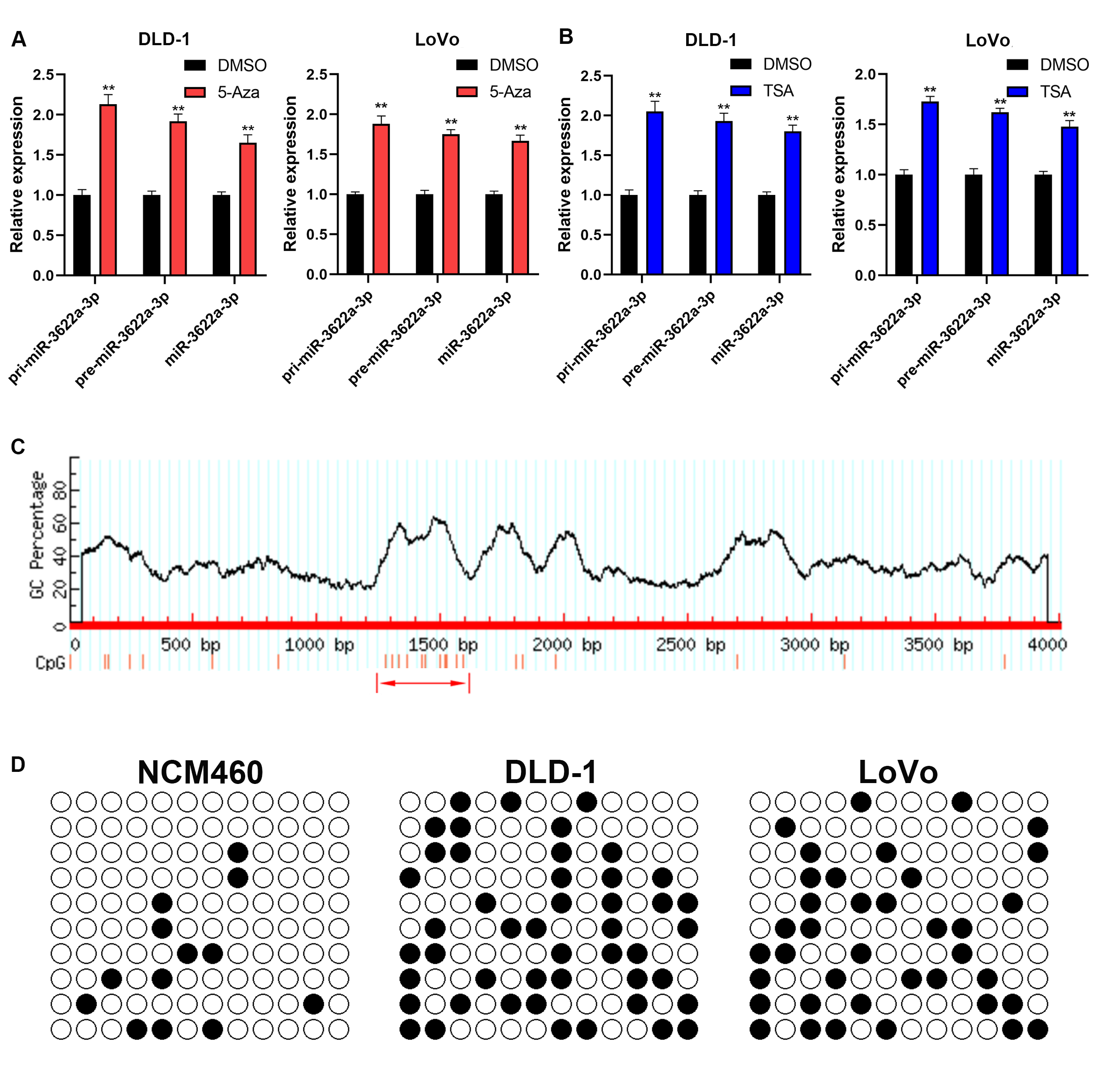

Supplement: Supplementary file 1 — Supplementary Figure 1 [file 41419_2020_2789_MOESM1_ESM.tif]

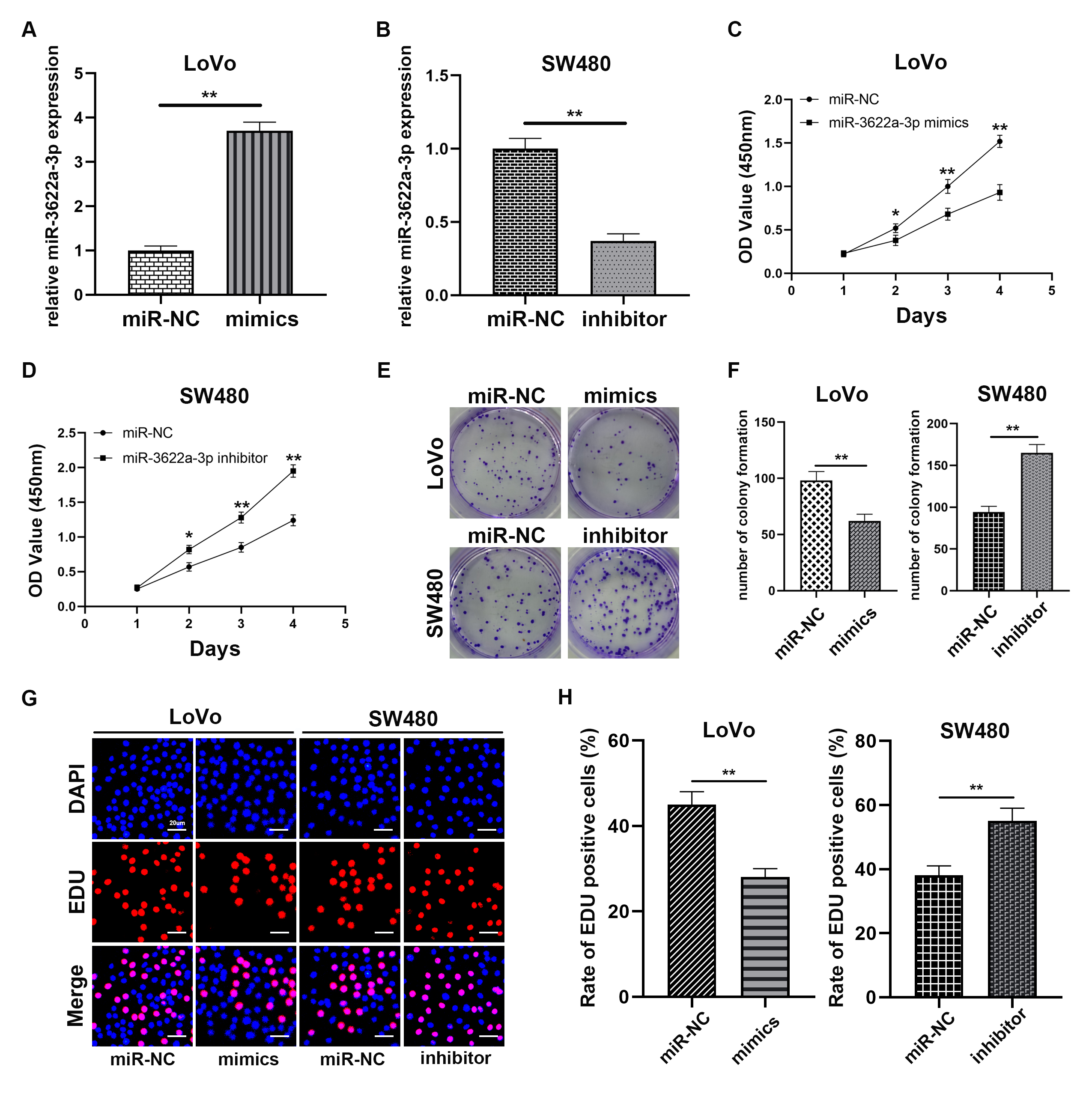

Supplement: Supplementary file 2 — Supplementary Figure 2 [file 41419_2020_2789_MOESM2_ESM.tif]

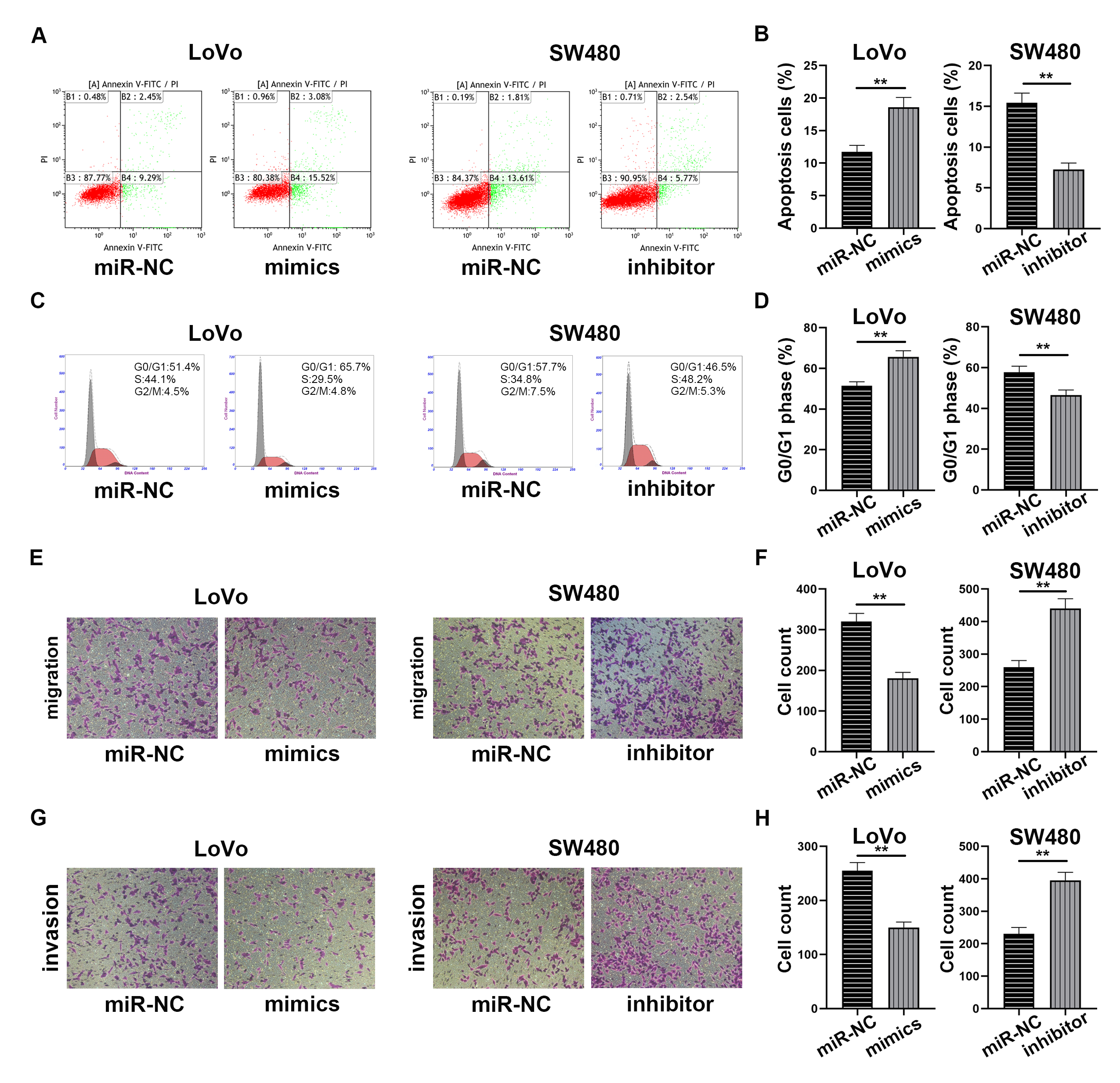

Supplement: Supplementary file 3 — Supplementary Figure 3 [file 41419_2020_2789_MOESM3_ESM.tif]

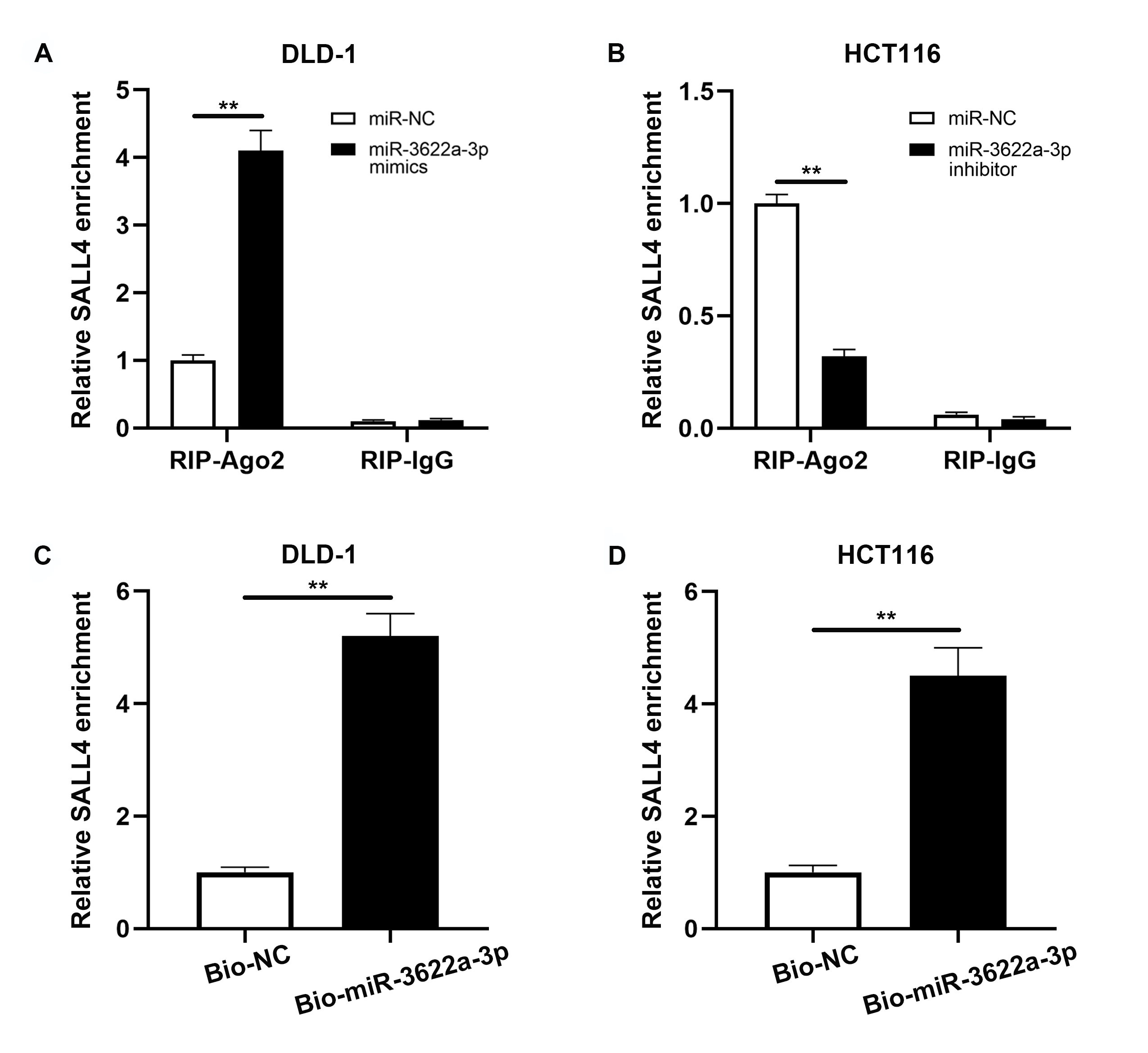

Supplement: Supplementary file 4 — Supplementary Figure 4 [file 41419_2020_2789_MOESM4_ESM.tif]

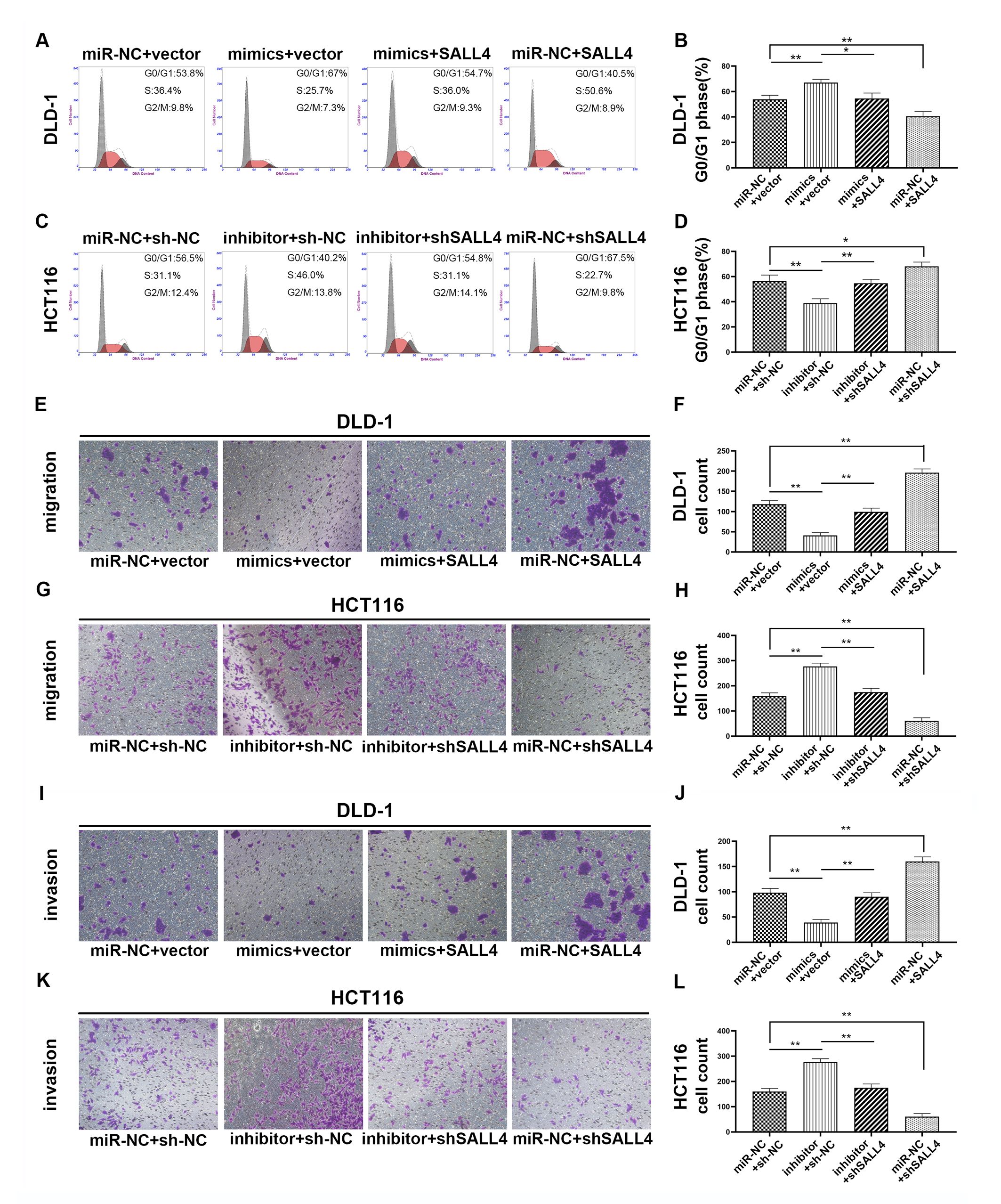

Supplement: Supplementary file 5 — Supplementary Figure 5 [file 41419_2020_2789_MOESM5_ESM.tif]
