# Supplementary material for: MiR-3622a-3p acts as a tumor suppressor in colorectal cancer by reducing stemness features and EMT through targeting spalt-like transcription factor 4
Source: Cell Death Dis. 2020 Jul 27;11(7):592. doi: 10.1038/s41419-020-02789-z (PMC7385142; doi:10.1038/s41419-020-02789-z)
Supplement: Supplementary file 6 — Supplementary Figure legend [file 41419_2020_2789_MOESM6_ESM.docx]

**Supplementary Figure Captions**

**Figure S1** **(A)** The relative expression levels of pri-miR-3622a-3p, pre-miR-3622a-3p and mature miR-3622a-3p in CRC cells were examined by qRT-PCR after incubation with 2.5μM 5-Aza for 72h. **(B)** The relative expression levels of pri-miR-3622a-3p, pre-miR-3622a-3p and mature miR-3622a-3p in CRC cells were examined by qRT-PCR after incubation with 100ng/ml TSA for 12h. **(C)** Scheme for the location of the CpG sites in the transcription start region of miR-3622a-3p. **(D)** The methylation of predicted CpG sites in NCM460, DLD-1 and LoVo cell lines was detected by the bisulfate sequencing method. Ten clones from each cell line were analyzed. All data are from three independent experiments and are presented as the means ± SD (*p < 0.05, **p < 0.01).

**Figure S2 (A)** Expression of miR-3622a-3p was increased in LoVo by miR-3622a-3p mimics transfection. **(B)** MiR-3622a-3p expression level was reduced in SW480 by miR-3622a-3p inhibitor transfection. **(C, D)** The effect of miR-3622a-3p on proliferation of LoVo and SW480 was evaluated by CCK-8 cell proliferation assay. **(E, F)** Colony forming ability of LoVo and SW480 was negatively corelated with miR-3622a-3p expression level. **(G, H)** The results of EDU assay suggested overexpression of miR-3622a-3p suppressed proliferation of LoVo while knockdown of miR-3622a-3p promoted SW480 proliferation. All data are from three independent experiments and are presented as the means ± SD (*p < 0.05, **p < 0.01).

**Figure S3 (A, B)** The effect of miR-3622a-3p on cell apoptosis of LoVo and SW480 was examined by flow cytometric analysis. **(C, D)** The effect of miR-3622a-3p on cell cycle of LoVo and SW480 was detected by flow cytometric analysis. **(E, F)** Transwell migration assay was performed to assess the influence of miR-3622a-3p on LoVo and SW480 migration abilities. **(G, H)** Transwell invasion assay was performed to assess the influence of miR-3622a-3p on LoVo and SW480 invasion abilities. All data are from three independent experiments and are presented as the means ± SD (*p < 0.05, **p < 0.01).

**Figure S4 (A, B)** RIP assay was performed to verify the interaction between miR-3622a-3p and SALL4 in DLD-1 and HCT116. **(C, D)** Pull-down assay was used to confirm that SALL4 is a target of miR-3622a-3p in DLD-1 and HCT116. All data are from three independent experiments and are presented as the means ± SD (*p < 0.05, **p < 0.01).

**Figure S5 (A-D)** Overexpression of SALL4 reduced the percentage of cells in G0/G1 phase and could reverse the effect of miR-3622a-3p on cell cycle of DLD-1. Knockdown of SALL4 induced G0/G1 cell cycle arrest and could reverse the effect of miR-3622a-3p knockdown on cell cycle of HCT116. **(E-H)** SALL4 played a promotive role in migration of CRC cells and the impact of miR-3622a-3p on CRC cell migration could be mediated by SALL4 regulation. **(I-L)** Overexpression of SALL4 contributed to invasion of CRC cells while down-regulation of SALL4 acted the opposite way. The influence of miR-3622a-3p on invasion of CRC cells was mediated by SALL4 regulation. All data are from three independent experiments and are presented as the means ± SD (*p < 0.05, **p < 0.01).
